# Evaluation of an mHealth tool to improve nutritional assessment among infants under 6 months in paediatric development clinics in rural Rwanda: Quasi‐experimental study

**DOI:** 10.1111/mcn.13201

**Published:** 2021-05-07

**Authors:** Mathieu Nemerimana, Angelique Charlie Karambizi, Sabine Umutoniwase, Dale A. Barnhart, Kathryn Beck, Vianney Kabundi Bihibindi, Kim Wilson, Alphonse Nshimyiryo, Jessica Bradford, Silas Havugarurema, Alphonsine Uwamahoro, Emmanuel Nsabyamahoro, Catherine M. Kirk

**Affiliations:** ^1^ Partners In Health/Inshuti Mu Buzima Kigali Rwanda; ^2^ Boston Children's Hospital Boston Massachusetts USA; ^3^ Kirehe District Hospital, Ministry of Health of Rwanda Kirehe Town Rwanda; ^4^ Harvard Medical School Boston Massachusetts USA

**Keywords:** child development, growth monitoring, high‐risk infants, mHealth, nutrition, small and sick newborns

## Abstract

Infants born preterm, low birthweight or with other perinatal complications require frequent and accurate growth monitoring for optimal nutrition and growth. We implemented an mHealth tool to improve growth monitoring and nutritional status assessment of high risk infants. We conducted a pre–post quasi‐experimental study with a concurrent control group among infants enrolled in paediatric development clinics in two rural Rwandan districts. During the pre‐intervention period (August 2017–January 2018), all clinics used standard paper‐based World Health Organization (WHO) growth charts. During the intervention period (August 2018–January 2019), Kirehe district adopted an mHealth tool for child growth monitoring and nutritional status assessment. Data on length/height; weight; length/height‐for‐age (L/HFA), weight‐for‐length/height (WFL/H) and weight‐for‐age (WFA) z‐scores; and interval growth were tracked at each visit. We conducted a ‘difference‐in‐difference’ analysis to assess whether the mHealth tool was associated with greater improvements in completion and accuracy of nutritional assessments and nutritional status at 2 and 6 months of age. We observed 3529 visits. mHealth intervention clinics showed significantly greater improvements on completeness for corrected age (endline: 65% vs. 55%; *p* = 0.036), L/HFA (endline: 82% vs. 57%; *p* ≤ 0.001), WFA (endline: 93% vs. 67%; *p* ≤ 0.001) and WFL/H (endline: 90% vs. 59%; *p* ≤ 0.001) z‐scores compared with control sites. Accuracy of growth monitoring did not improve. Prevalence of stunting, underweight and inadequate interval growth at 6‐months corrected age decreased significantly more in the intervention clinics than in control clinics. Results suggest that integrating mHealth nutrition interventions is feasible and can improve child nutrition outcomes. Improved tool design may better promote accuracy.

**Key messages**

- We evaluated whether using an mHealth tool can improve completeness and accuracy of growth monitoring and nutritional status assessment among at‐risk infants enrolled in a paediatric development clinic in rural Rwanda.
- Integrating the mHealth tool for growth monitoring and nutritional interventions was feasible and was associated with significantly greater improvements in completeness of growth monitoring but not improvements in growth monitoring accuracy.
- The mHealth tool was associated with reductions of malnutrition at 6 months suggesting that mHealth tools can help improve nutritional outcomes among high‐risk infants; however, additional design changes may be required to promote accuracy.

​

## 1 INTRODUCTION

Globally, tremendous improvement has been made during the past decades in newborn care and reduced neonatal mortality and morbidity, especially in low‐ and middle‐income countries (Darmstadt et al., 2015). As more at‐risk newborns survive, the burden of disability secondary to newborn conditions in these countries has increased (Lawn et al., 2014). Infants born with perinatal complications such as prematurity, low birthweight (LBW) and neonatal encephalopathy are at high‐risk for acute malnutrition, stunting and feeding difficulties leading to adverse developmental outcomes (Danaei et al., 2016; Kerac et al., 2014). Early identification and interventions for nutritional problems can help in preventing malnutrition and improving children's growth and development (Valérie et al., 2010).

Optimal nutrition and growth of vulnerable children requires close appropriate growth monitoring through regular follow‐up. The effectiveness of child growth monitoring depends both on the accuracy of measurements of weight, length/height, interval growth and head circumference as well as correctly using these measurements to identify nutrition problems. World Health Organization (WHO) growth charts are often used to assess child growth through calculation of z‐scores, including weight‐for‐length/height (WFL/H), weight‐for‐age (WFA), length/height‐for‐age (L/HFA) and head circumference‐for‐age among children under five (World Health Organization [WHO], 2006). However, challenges among health care providers in using child growth charts have been reported, including misinterpretation of growth trajectories, poor plotting, inappropriate growth classifications, wrong rating of weight gain and under‐consideration of slow weight gain in taking actions (Bradford et al., 2020; Ezeofor et al., 2017; Mutoro & Wright, 2013). The possibility of incorrect classification and decision is even greater among preterm born infants, for whom a corrected age must be calculated prior to using the WHO growth charts (Villar et al., 2018).

mHealth interventions, defined as use of any mobile technology such as mobile phones and tablets in health (Zapata et al., 2015), may help reduce inaccuracies in growth monitoring and minimize errors in decision making (Chanani et al., 2016). Various mHealth tools have been successfully applied to improve delivery of health services in different health contexts (Bonilla et al., 2015; Källander et al., 2013; Mechael et al., 2010; Pop‐Eleches et al., 2011). The use of an mHealth mobile calculator in India previously showed positive effects in improving accuracy in screening for malnutrition and reduction of errors in child growth classification among frontline health workers (Chanani et al., 2016), and in Indonesia, a mobile application has improved accurate classification of growth status (Barnett et al., 2016). However, little is known on the effectiveness of mHealth apps in monitoring growth of high‐risk infants.

Partners In Health/Inshuti Mu Buzima in collaboration with the Rwanda Ministry of Health developed the paediatric development clinic (PDC) to provide structured follow up for high‐risk infants including infants born premature, LBW, or with other perinatal complications. The PDC programme is integrated into district hospitals and primary‐level community health centres to help high‐risk infants survive and achieve their full potential by providing comprehensive health, nutritional and developmental care with prevention, early detection of risks or complications and provision of specific interventions to identified problems (Ngabireyimana et al., 2017). PDC services are delivered by nurses and social workers supported by general practitioners. To help infants achieve good nutritional outcomes, nurses provide nutritional care through growth monitoring, counselling and interventions such as supplementary foods for mothers to support breastfeeding or infant formula when medically necessary. However, challenges such as difficulties among providers in calculating corrected age, interval growth and correct plotting on WHO growth charts were identified (Beck et al., 2018; Bradford et al., 2020). An mHealth application tool on an Android tablet was developed with the objective to support nurses in completing accurate child growth classification; decision making aligned with the nutrition protocol; and early identification of inadequate interval growth, WFL/H, WFA and L/HFA z‐scores. The aims of this study were to evaluate whether using a mHealth tool improved (1) completeness of growth monitoring assessment of at‐risk infants; (2) accuracy of providers' interpretations of growth monitoring; and (3) the nutritional status of infants enrolled in PDCs at 2 and 6 months.

## 2 METHODS

### 2.1 Study design

A pre–post quasi‐experimental design with a nonmatched comparison group was used to compare the quality of infant growth monitoring among infants enrolled in PDCs. Infants enrolled in PDCs using an mHealth tool to support nurses' infant growth monitoring were compared with other infants enrolled in PDCs using standard paper‐based growth monitoring tools. The study was composed of two phases, the pre‐intervention period from August 2017 to January 2018 and the post‐intervention period from August 2018 January 2019. Kirehe district PDCs implemented the mHealth intervention at the end of the pre‐intervention period and served as our intervention group while, Kayonza district PDCs were used as a nonmatched control districts. The number of infants referred to PDCs varies depending of infants identified and meeting referral criteria and the number of referral changes. The chosen study design was selected to allow continuous provision of full package services to all children in PDC.

### 2.2 Study setting

This study was conducted in PDCs at two rural district hospitals, Rwinkwavu and Kirehe, and seven health centres located in the catchment areas of these hospitals. These health centres are Kabarondo, Cyarubare, Ruramira and Ndego, which are located in the Rwinkwavu catchment area in Kayonza district, and Musaza, Gahara and Mushikiri health centres, which are located in the Kirehe catchment area. These are public health facilities under the Rwandan Ministry of Health and are located in rural settings in the Eastern Province of Rwanda. Together, Rwinkwavu and Kirehe district hospitals and affiliated health centres serve a total population of over 600,000 people (National Institute of Statistics of Rwanda [NISR] [Rwanda] & Ministry of Finance and Economic Planning [MINECOFIN] [Rwanda], 2012). PDCs operate as outpatient clinics.

The PDC programme was initially started at Rwinkwavu District Hospital in April 2014 and was expanded to Kirehe District Hospital in May 2016. Most infants admitted to PDCs are referred from the hospital inpatient neonatal care units. The main reasons for referral and admission to PDC are prematurity, LBW < 2000 g, hypoxic ischemic encephalopathy (HIE), cleft lip/palate and trisomy 21. Other reasons for admission include developmental delay, hydrocephalus and infants aged less than 12 months following hospitalization for severe acute malnutrition. These high‐risk infants are followed up until age five.

Details on PDC programme and protocol have been described elsewhere (Beck et al., 2018; Ngabireyimana et al., 2017). PDC services are primarily provided by trained nurses and social workers, receiving support from general practitioner. PDC health providers receive an intensive 5‐days training on the PDC medical, nutrition and developmental care protocol as well as on general principles of early childhood development before they start to provide the services. All children enrolled in PDC are followed up with the standard visits at 1 week, 2 weeks, 3 weeks, 1 month, 2 months, 4 months, 6 months, 9 months and 12 months of age, then the follow‐up is continued every 6 months. At each visit, children are provided PDC interventions package that include group counselling, routine clinical check‐ups, developmental and responsive care, enhanced nutrition care for small and sick newborns and integrated social support. Group counselling sessions provide space for parents to get peer support, and parents are educated on different health, nutrition and child development topics. Routine clinical check‐ups include screening for danger signs, health complications or risks and medical interventions that include referral are provided to identified issues and according to child conditions. Developmental care includes developmental monitoring, group session on play and communication, individual counselling and coaching of parents on responsive caregiving. Children identified with developmental difficulties or disabilities are referred for early intervention.

For enhanced nutrition care, growth monitoring and feeding assessments are conducted to determine nutritional status of the child. A nurse assesses the child's anthropometric measurements, calculates interval growth and plots WFL/H, WFA and L/HFA z‐scores on WHO growth charts. Based the plotted charts, the nurse classifies the growth of the child and determines the child nutrition status. For feeding assessment, the nurse assesses the breastfeeding, feeding patterns and feeding difficulties. Food packages are provided to nutritionally at‐risk infants, and children identified with malnutrition are referred to malnutrition management programme at the health centre. Parents are provided with nutritional and feeding counselling based on the child's nutritional status and feeding pattern. PDC social worker conducts social risk assessment, determines social support needed, provides individual counselling for psychosocial support and collaborates with the nurse to address social needs of the child and parents. Through referral system, PDCs are linked with other health facility clinical services. If the child is identified with acute illness or severe malnutrition with complications or any other medical complications, he/she is referred for inpatient care and/or medical review.

During the pre‐intervention period, nurses at all clinics were using paper‐based charts for child growth monitoring and for assessing nutrition status. Nurses used the 2006 WHO growth charts to plot child growth z‐scores and recorded their interpretations into the patients' files.

### 2.3 Study population

We assessed accuracy and completion of growth monitoring among all visits occurring during the pre‐intervention (August 2017–January 2018) and post‐intervention periods (August 2018–January 2019), regardless of the child's age. We assessed nutritional status at age 2 and 6 months (corrected for prematurity) among infants less than 6 months who enrolled in PDCs in Kirehe and Kayonza districts during the study period (August 2017–January 2019). Although is it possible for children to have their growth assessed at other ages, for example, if they present for a nonemergency acute illness, these ages were chosen purposively because they presented early growth (2 months) and the oldest age possible with our dataset (6 months) as a measure of longer term follow‐up. In addition, PDC programme systematically conducts growth assessments at ages 2 and 6 months such that we could expect to have complete data for most infants enrolled in the programme for at least 2 or 6 months at these ages. We included infants who have been enrolled before 2 months of age (corrected for prematurity) and had at least one PDC visit before or after 1 month of the target age for nutritional assessment (2 and 6 months). For all analyses, we excluded any post‐intervention visits from infants who initially enrolled during pre‐intervention period, but whose treatment continued during intervention period. This exclusion criterion was selected to avoid contamination of study groups. Our retrospective analysis included data on all eligible infants during the study period.

### 2.4 Intervention description

An mHealth tool was developed by Partners In Health/Inshuti Mu Buzima in collaboration with DTree International and ThingsPrime to support PDC health providers in conducting growth and nutritional assessments of PDC patients with the goal of improving early identification of growth failure and supporting provider decision making and counselling aligned with the protocol for the PDC programme. The mHealth tool is an android tablet/smartphone‐based application built using MangoLogic software with algorithms for growth monitoring and decision support that are based on the 2006 WHO Child Growth Standards and additional clinical information, including gestational age, based on PDC protocol for nutritional management of high risk infants. The tool is automated to plot child's anthropometric z‐scores and to calculate interval weight gain and automatically provides quick growth classification represented using colour‐coded visual pictures (green for normal nutritional status, yellow for moderate malnutrition and red for severe malnutrition). The tool provides final decision on nutritional status and guiding messages for the health provider to plan nutritional interventions for the child. The function for mHealth tool to record entries was deactivated, and the tool does not track patient identifiers and entered measurements.

To use the tool, the health care provider enters the patient's anthropometric data (length/height and weight), gestational age at birth and birthdate into the mHealth tool. The mHealth tool is programmed to calculate chronological age and corrected age for patients born premature (<37 weeks), as well as WFL/H, WFA and L/HFA z‐scores, and interval growth (average daily weight gain since last measurement). To aid interpretation of the calculations, we programmed the tool to determine the nutritional status of the patient according to the PDC protocol and provide a counselling message related to the patient's nutritional status, such as encouraging praise and counselling on play and communication when a child is growing well. The PDC provider uses the results and guiding messages provided by the mHealth tool to develop individualized nutrition care plans for the child and provides face to face nutritional counselling to the parents to address any specific concerns using additional counselling support in the PDC job aid.

Prior to disseminating the tool to PDC health care workers, the mHealth tool went through several rounds of testing by Partners In Health/Inshuti Mu Buzima programme staff to validate the recommendations from the tool against the PDC protocol and standard WHO growth charts. The Android‐supported mHealth tool was installed onto tablets. Seven PDC health providers (four nurses and three social workers) from three health facilities with PDC in Kirehe district received a 2‐day training to learn how to use the tablet and tool through several case study‐based practical exercises. All health providers had experience in using personal android smartphones. Following the training, one tablet per health facility was distributed to each Kirehe district PDC and was primarily used by four trained nurses during clinical consultation. Health providers used the mHealth tool to conduct nutritional assessments and documented results from the tool (corrected age, z‐scores and interval growth) on hard copy clinic visit forms that were later entered into an electronic medical record (EMR). Health providers received on‐going mentorship on the use of the tool in the clinics by trained nutritionist and PDC programme manager from Partners In Health/Inshuti Mu Buzima as part of routine clinic mentorship. Nurses in the Kayonza district PDCs continued to use paper‐based growth monitoring. In our study sites, there was no additional formal training on the use of paper growth charts and interpretation, beyond overall PDC protocol training, which was attended by all PDC health care providers in in September 2017.

### 2.5 Data collection

In both intervention and control sites, data were extracted from the EMR, which uses an OpenMRS system to record patients' information. Nurses routinely documented the clients' clinical information in patients' paper files, and that information was entered into the EMR by trained data officers within 1 week of the visit. Data on sociodemographic characteristics, anthropometric measurements, nurse‐reported child growth z‐scores (WFL/H, WFA, L/HFA), and nutritional status for the infants meeting inclusion criteria were extracted from the EMR. Data were deidentified prior to analysis. Monthly routine data quality assessments and validations are conducted regularly by district EMR coordinators with immediate correction of errors identified, and data were assessed for implausible values during the analysis process.

### 2.6 Data analysis

Data from the EMR records were assessed for plausibility and extreme values were flagged as data entry errors, investigated, and corrected, whenever possible. Our primary unit of analysis was PDC visits, which were cross‐classified into four exposure groups based on whether they occurred at either control vs. intervention sites during either the pre‐intervention or post‐intervention periods. The four exposure groups were (1) control pre‐intervention, (2) control post‐intervention, (3) intervention pre‐intervention and (4) intervention post‐intervention. We described the characteristics of PDC visits across exposure groups by calculating proportions for categorical variables and medians and interquartile ranges (IQRs) for continuous variables. A child was defined as preterm if their gestational age at birth was less than 37 weeks or gestational age was missing but the child was referred to the PDC clinic as preterm. Corrected age was calculated for any preterm infant with a documented gestational age as the infant's chronological age minus the number of weeks born premature, where weeks born early is defined as 40 weeks minus gestational age in weeks. LBW was defined as weight at birth less than 2500 g, though criteria for enrolment in PDCs match the criteria for referral of an infant born LBW to a hospital inpatient neonatal unit, which is less than 2000 g. Small for gestational age (SGA) was defined as birthweight less than the 10th percentile for gestational age and was calculated using INTERGROWTH‐21st standards (Villar et al., 2014).

To assess the completeness of growth monitoring conducted at PDCs, we calculated the proportion of visits where nurses recorded responses for infants' length, weight, categorized L/HFA z‐score, categorized WFL/H z‐score, categorized WFA z‐score and interval growth across exposure groups. Because the axes of the WHO growth charts do not extend beyond certain values, in some cases, nurses were instructed to record the nutritional assessment as ‘not applicable’ (see Table 1 for details). Therefore, responses of ‘not applicable’ were considered to be complete. Interval growth was considered complete if it was recorded as either a continuous (e.g., 10 g/day) or categorical (e.g. ‘adequate growth’) variable. Among preterm infants, we also assessed the proportion of visits where nurses reported a corrected age.

**TABLE 1:** Standardized definitions

	Normal/adequate	Malnourished/inadequate		Not applicable
		Moderate	Severe	
L/HFA z‐score	≥ −2	< −2 and ≥ −3	< −3	Corrected ages is <0 days OR length/height is <42 cm
WFL/H z‐score	≥ −2	< −2 and ≥ −3	< −3	Length/height is <45 cm OR corrected age is <0 days OR weight <1.6 kg
WFA z‐score	≥ −2	< −2 and ≥ −3	< −3	Corrected age is <0 days OR weight<1.4 g
Interval growth	≥20 g/day if corrected age <3 mo ≥15 g/day if 3 mo ≥ corrected age <6 mo ≥10 g/day if 6 mo ≥ corrected age <8 mo ≥6 g/day if 8 mo ≥ corrected age <12 mo ≥5 g/day if 12 mo ≥ corrected age <16 mo ≥4 g/day if 16 mo ≥ corrected age <24 mo	<20 g/day if corrected age <3 mo <15 g/day if 3 mo ≥ corrected age <6 mo <10 g/day if 6 mo ≥ corrected age <8 mo <6 g/day if 8 mo ≥ corrected age <12 mo <5 g/day if 12 mo ≥ corrected age <16 mo <4 g/day if 16 mo ≥ corrected age <24 mo		Corrected age ≥24 mo or weight at last visit is missing

Abbreviations: L/HFA, length/height‐for‐age; mo, months; WFA, weight‐for‐age; WFL/H, weight‐for‐length/height.

To assess the accuracy of growth monitoring, we used recorded data on length/height, weight, age and gestational age at birth to characterize infants' nutritional status at each visit according to standardized WHO definitions using the WHO's iGrowUp macros in Stata (Table 1). For each nutritional status assessment completed by PDC nurses, we evaluated the concordance between PDC nurse growth classifications and the child's nutritional status based on these standard definitions across exposure groups. For L/HFA z‐scores, WFL/H z‐scores and WFA z‐scores, visits were classified as concordant if nurse‐recorded categories of ‘normal (z‐score ≥ −2)’, ‘moderate malnutrition (z‐score < −2)’, ‘severe malnutrition (z‐score < −3)’ or ‘not applicable’ corresponded to the standardized definitions. Interval growth was calculated as the average weight gain between two weight measurements. It is calculated by taking the weight of the child today minus the prior weight of the child and dividing by the number of days between the two visits. For interval growth, we excluded each child's first visit from analysis because weight at discharge from birth facility, the variable most commonly used to calculate interval growth during the first visit to a PDC, was not available in the EMR. Interval growth entries were classified as concordant if nurse‐recorded categories of ‘adequate’, ‘inadequate’ or ‘not applicable’ corresponded to standard classifications or, in cases where interval growth was recorded as continuous but not categorized by the nurse, if the reported continuous interval growth value would have enabled accurate classification of a child's growth as adequate vs. inadequate. PDC nurse growth classifications were classified as discordant if (1) nurse‐recorded categories did not correspond to standardized definitions, (2) nurses did not record sufficient data to validate their response, or (3) for z‐score‐based metrics, nurses recorded implausible length or weight data such that the child's z‐score was ±5 standard deviations beyond the mean.

To assess whether the mHealth intervention led to overall improvements in infants' nutritional status, we assessed the prevalence of stunting, wasting, underweight and inadequate growth across exposure groups among 2‐ and 6‐month‐old infants (corrected age). Stunting was defined as L/HFA z‐score of < −2; wasting was defined as WFL/H z‐score < −2; and underweight was defined as WFA z‐score < −2. Interval growth was categorized based on PDC protocol as adequate or in adequate depending on the child's corrected age (Table 1). This analysis was conducted at the child level using data from infants' closest visit within 1 month of the target date. Infants without a visit within 1 month of the target date or with incomplete or implausible data on length and weight were excluded from the analysis.

To test whether intervention sites experienced greater improvements in growth monitoring completion, growth classifications accuracy and infants' nutritional status over time relative to control sites, we used a linear regression to conduct a ‘difference‐in‐difference’ analysis (Dimick & Ryan, 2014). The use of linear regression with binary outcomes and robust standard errors allowed coefficients from regression models to be interpreted as percentages (Cheung, 2007), and we accounted for clustering among visits occurring at the same facility (Mansournia et al., 2020; Rogers, 1993). Because the distribution of PDC visits occurring at hospitals vs. health centres changed over the study period, especially in the intervention group, we conducted a sensitivity analyses in which we adjusted our regression models for health facility type. Adjusting for this potential confounding by PDC visit location did not substantively alter our interpretations of the model parameters.

### 2.7 Ethics

The study was approved by the Rwanda National Ethics Committee and was conducted as part of the PDC evaluation. Because this study was a retrospective analysis of existing clinical data, Rwanda National Ethics Committee waived the requirement for written consent.

## 3 RESULTS

Our analysis included data from 3529 visits from 880 children (see Data S1 for consort diagram). Table 2 describes the visit characteristics and demographic and clinical traits of children from PDCs from the pre‐intervention and post‐intervention periods in both comparison and intervention clinics. In the comparison clinics, the majority of visits occurred in health centres during both the pre‐intervention period (71%) and the post‐intervention period (75%). In the intervention clinics, the introduction of the mHealth intervention coincided with decentralization, with 9% of visits occurring at health centre during the pre‐intervention period and 43% occurring at health centres during the post‐intervention period. Otherwise, the populations served across districts are similar during the pre‐intervention and post‐intervention periods. The majority of infants' mothers had no formal education and were older than 30 years. Across both intervention and comparison groups in both periods, the majority of infants were diagnosed as preterm or with LBW, with about one‐fifth of infants being diagnosed with HIE. Relatively few infants were diagnosed with other or multiple conditions.

**TABLE 2:** Demographic and clinical characteristics of visits and children enrolled in PDCs in the comparison and intervention clinics during the pre‐intervention period and post‐intervention period

	Comparison clinics (*n* = 1566)				Intervention clinics (*n* = 1963)			
	Pre‐intervention		Post‐intervention		Pre‐intervention		Post‐intervention	
	*n*	%	*n*	%	*n*	%	*N*	%
Visit characteristics (*N* = 3529)	866		700		1051		912	
Location/clinic of enrolment								
Health centre PDC	618	71	523	75	93	9	389	43
Hospital PDC	248	29	177	25	958	91	523	57
Child's age in months, corrected for prematurity (median, IQR)	1.8	(0.5, 4.4)	2.3	(0.8, 5.3)	1.5	(0.3, 5.4)	1.6	(0.4, 5.2)
Child characteristics at birth (*N* = 880)								
Caregiver level of education	214		151		288		227	
No formal education completed (<P6)	104	49	72	48	119	41	135	60
Primary school completed (≥P6 and <S6)	61	29	43	29	77	27	53	23
Secondary school completed (≥S6)	2	0.9	1	0.7	14	5	12	5
Missing	47	22	35	23	78	27	27	11.9
Age of mother at child's enrolment in PDC								
<20 years	17	8	13	9	13	5	9	4
20–24 years	54	25	30	20	61	21	37	16
25–29 years	39	18	27	18	76	26	59	26
30 + years	81	38	53	35	123	43	103	45
Missing	23	11	28	19	15	5	19	8
Household socioeconomic category (Ubudehe)								
Category 1	23	11	15	10	27	9	29	13
Category 2	76	36	46	31	114	40	88	39
Category 3	91	43	60	40	74	26	67	30
Category 4	0	0.0	0	0.0	1	0.3	2	0.9
Missing	24	11	30	19.9	72	25	41	18
Number of children in household (median, IQR)	3	(1, 4.5)	2	(1, 4)	3	(2, 4)	2	(1, 4)
Child's sex								
Male	117	55	72	48	140	49	125	55
Female	97	45	79	52	148	51	102	45
Prematurity								
No (≥37 weeks)	90	42	84	56	124	43	91	40
Yes (<37 weeks)	124	58	67	44	164	57	136	60
Child's weight at birth								
<1500 g	29	14	12	8	35	12	20	9
1500–1999 g	54	25	41	27	84	29	62	27
2000–2499 g	44	21	21	14	49	17	42	19
≥2500 g	49	23	29	19	83	29	61	27
Missing data	38	18	48	32	37	13	42	19
Small for gestational age								
No	67	31	37	25	101	35	73	32
Yes	70	33	35	23	95	33	70	31
Missing data	77	36	79	52	92	32	84	37
Child diagnosed as preterm or low birthweight								
No	65	30	65	43	89	31	74	33
Yes	149	70	86	57	199	69	153	67
Child diagnosed with HIE								
No	162	76	120	80	225	78	176	78
Yes	52	24	31	21	63	22	51	23
Child diagnosed with other conditions^a^								
No	195	91	140	93	254	88	205	90
Yes	19	9	11	7.3	34	11.8	22	9.7
Child diagnosed with multiple conditions								
No	190	89	144	95	265	92	207	91
Yes	24	11	7	5	23	8	20	9

^a^ Other conditions include the following: central nervous system infections, trisomy 21, post‐hospitalization for severe malnutrition, hydrocephalus, cleft lip or palate and other developmental delays.

Abbreviations: HIE, hypoxic ischemic encephalopathy; IQR, interquartile range; PDC, paediatric development clinic.

Table 3 describes the completeness of growth monitoring and nutritional status assessment among child visits in the PDCs at mHealth intervention clinics and comparison clinics during the pre‐ and post‐intervention. Except for corrected age, the completion of nutritional status recording during the pre‐intervention period was similar in both groups. Weight and length/height recording was consistently high in both groups across all periods (99%–100%). After mHealth intervention, the completion of corrected age (55% at control sites vs. 65% at intervention sites; *p* = 0.036), L/HFA z‐score assessment (57% at control sites vs. 82% at intervention sites; *p* < 0.001), the WFA z‐score assessment (67% at control sites vs. 93% at intervention sites; *p* < 0.001) as well as the WFL/H z‐score assessment (59% at control sites vs. 90% at intervention sites; *p* < 0.001) had improved significantly more at intervention sites compared with control sites.

**TABLE 3:** Completeness of growth monitoring among child‐visits in the PDCs at intervention and comparison clinics during the pre‐intervention period and post‐intervention period

	Comparison clinics (*n* = 1566)				Intervention clinics (*n* = 1963)				
	Pre‐intervention (*N* = 866)		Post‐intervention (*N* = 700)		Pre‐intervention (*N* = 1051)		Post‐intervention (*N* = 912)		
	*n*	%	*n*	%	*n*	%	*n*	%	*p* value
Weight recorded by nurse									0.697
No	2	0.2	3	0.4	2	0.2	2	0.2	
Yes	864	100	697	100	1049	100	910	100	
Length recorded by nurse									0.295
No	6	0.7	4	0.6	2	0.0	5	0.6	
Yes	860	99	696	99	1049	100	907	99	
Corrected age calculated by nurse^a^									0.036
No	226	49	153	45	534	94	205	35	
Yes	274	51	186	55	32	6	379	65	
Length for age z‐score assessed by nurse^b^									<0.001
No	201	23	162	43	226	22	129	18	
Yes	665	77	212	57	825	79	575	82	
Weight for length z‐score assessed by nurse									<0.001
No	209	24	285	41	234	22	94	10	
Yes	657	76	415	59	817	78	818	90	
Weight for age z‐score assessed by nurse									<0.001
No	199	23	234	33	221	21	62	7	
Yes	667	77	466	67	830	79	850	93	
Interval growth assessed by nurse									0.115
No	164	19	136	19	194	18	88	10	
Yes	702	81	564	81	857	82	824	90	

^a^ Among preterm infants only (*N* = 2029).

^b^ Starting 1 March 2019, paediatric development clinic (PDC) nurses began to record LFA‐z‐scores at standard growth assessment visits, rather than at all PDC visits, which included urgent care visits. We have restricted this analysis to visits occurring before 1 March 2019 and standard growth assessment visits occurring after 1 March 2019 (*N* = 2995).

The concordance of classification of the child's nutritional status and interval growth by health care providers using either mHealth tool or paper‐based WHO growth charts and gold standard calculations with Stata is described in Table 4. In pre‐intervention, the levels of agreement between health care provider's classification and the gold standard calculations in Stata‐based classification ranged between 77% and 90% for intervention clinics and 64% and 77% for comparison clinics. In post‐intervention, the concordance ranged from 72% to 87% in intervention clinics and from 64% to 75% in comparison clinics. There was no significant difference in the change in concordance between comparison and intervention clinics.

**TABLE 4:** Concordance of growth monitoring between paediatric development clinic (PDC) nurses and World Health Organization (WHO) standard growth charts before and after the introduction of an mHealth tool

	Comparison clinics				Intervention clinics				
	Pre‐intervention		Post‐intervention		Pre‐intervention		Post‐intervention		
	*n*	%	*n*	%	*n*	%	*n*	%	*p* value*
Concordance in interpretation of length‐for‐age z‐score									0.434
Not matching	242	36	76	36	188	23	161	28	
Matching	423	64	136	64	637	77	414	72	
Concordance in interpretation of weight‐for‐length z‐score									0.436
Not matching	174	26	105	25	125	15	141	17	
Matching	483	74	310	75	692	85	677	83	
Concordance in interpretation of weight‐for‐age z‐score									0.544
Not matching	217	33	168	36	159	19	209	25	
Matching	450	67	298	64	671	81	641	75	
Concordance in interpretation of interval growth^a^									0.617
Not matching	125	23	124	28	63	10	82	13	
Matching	428	77	321	72	554	90	543	87	

* *p* value for ‘difference‐in‐difference’ analysis assessing whether the pre‐ to post‐intervention change in concordance between PDC nurse classification and WHO standards differed among intervention vs. control sites.

^a^ First visits from the PDC were excluded from concordance analysis because electronic medical record (EMR) records did not include infants' weight at discharge from birth facility.

Table 5 describes the nutritional status and interval growth of infants receiving the mHealth intervention and of those not receiving the intervention at 2 and 6 months of age. Among the 460 infants assessed at 2‐months corrected age, there were no significant differences in the changes of the prevalence of stunting, wasting, underweight and inadequate interval growth from pre‐ to post‐intervention in both intervention and comparison clinics. Among the 158 infants assessed at 6‐months corrected age, stunting in intervention clinics declined from 59% to 44%, whereas it increased from 48% to 58% in comparison clinics (*p* = 0.041), in pre‐ to post‐intervention, respectively. Underweight decreased from 47% to 33% in intervention clinics and increased from 30% to 53% in comparison clinics (*p* = 0.014). The proportion of infants with inadequate interval growth fell from 59% to 26% in intervention clinics, whereas it increased from 52% to 56% in comparison clinics (*p* < 0.001). In addition, the prevalence of wasting declined from 19% to 10% in intervention clinics, whereas it increased from 18% to 21% in comparison clinics, though the difference was not statistically significant (*p* = 0.366).

**TABLE 5:** Nutritional status and interval growth of infants receiving the mHealth intervention and of those not receiving the intervention at 2 and 6 months of age

	Comparison clinics				Intervention clinics				*p* value*
	Pre‐intervention		Post‐intervention		Pre‐intervention		Post‐intervention		
	*n*	%	*n*	%	*n*	%	*n*	%	
Infants at 2‐months corrected age (*N* = 460)^a^									
Stunting (length‐for‐age)									
Normal (z‐score ≥ −2)	66	53	46	50	68	52	66	59	0.497
Stunted (z‐score < −2)	58	47	47	51	64	48	45	41	
Wasted (weight‐for‐length)									0.098
Normal (z‐score ≥ −2)	106	85	85	91	120	91	98	88	
Wasted (z‐score < −2)	18	15	8	9	12	9	12	12	
Underweight (weight‐for‐age)									0.927
Normal (z‐score ≥ −2)	71	57	49	53	78	59	61	55	
Underweight (z‐score < −2)	53	43	44	47	54	41	50	45	
Interval growth^b^									
Adequate	88	73	69	77	107	82	84	76	0.233
Inadequate	32	27	21	23	24	18	26	24	
Infants at 6‐months corrected age (*N* = 158)^a^									
Stunting (length‐for‐age)									
Normal (z‐score ≥ −2)	23	52	18	42	13	41	22	56	0.041
Stunted (z‐score < −2)	21	48	25	58	19	59	17	44	
Wasted (weight‐for‐length)									0.366
Normal (z‐score ≥ −2)	36	82	34	79	26	81	35	90	
Wasted (z‐score < −2)	8	18	9	21	6	19	4	10	
Underweight (weight‐for‐age)									0.014
Normal (z‐score ≥ −2)	31	70	20	47	17	53	26	67	
Underweight (z‐score < −2)	13	30	23	53	15	47	13	33	
Interval growth^b^									0.001
Adequate	21	48	19	44	13	41	29	74	
Inadequate	23	52	24	56	19	59	10	26	

* *p* value for ‘difference‐in‐difference’ analysis assessing whether the pre‐ to the post‐intervention change in percentage of infants with malnutrition (moderate or severe) differed among intervention vs. control sites.

^a^ Analysis restricted to infants' closest visit ±1 month of the target date among infants who had enrolled before 2 months of age. Observations with incomplete or implausible data on length and weight were excluded from analysis.

^b^ Excludes infants with missing weight at last analysis (*N* = 451).

## 4 DISCUSSION

The results of this study demonstrate a strong association between the introduction of an mHealth tool and improvements in the completeness in growth monitoring among care providers. The use of mHealth tool was also associated with significantly greater reductions in malnutrition among 6‐month old infants at sites receiving the mHealth intervention compared with those at sites not receiving the intervention. However, we did not find any improvements in accuracy associated with the introduction of the mHealth tool.

In our study, there was a significant improvement of level of completeness at intervention clinics. The mHealth tool provides automatic calculation of corrected age, anthropometric z‐scores, and interval growth with a quick representation of classification of child nutritional status and guiding messages for clinical decisions. These features may have motivated the nurses to complete all aspects of the nutritional assessment, especially for harder‐to‐calculate growth classifications. The nurses in the PDCs reported that they appreciated the use of the mHealth tool, suggesting that the implementation of this tool may be feasible elsewhere. A similar finding is found in a study done in New Zealand where introduction of use of electronic growth charts at hospital was found to increase the rates of recording growth measurements and body mass index z‐scores (Dainty et al., 2019).

Even though the accuracy of the nurses' documented growth classifications did not improve, the improvement in the completeness of nutritional assessments at the same level of accuracy may have been sufficient to generate meaningful improvements in quality of care. More complete growth monitoring may have increased the number of parents who received targeted nutritional counselling. We did not observe any improvements in the prevalence of malnutrition among infants at age 2 months, perhaps because it can take several months of intervention to improve nutritional status. However, we did observe significantly larger reductions in the prevalence of malnutrition among infants at age 6 months in clinics with the mHealth intervention compared with clinics without the intervention. This finding suggests that, even in the absence of improved accuracy, the increased completion associated with the mHealth intervention may have been sufficient to improve nutritional outcomes. This finding also suggests that quality improvements in nutritional interventions such as the PDC program may take several months to result in observable changes in nutritional status.

Our intervention was not associated with an improvement in growth monitoring accuracy. Improving growth monitoring interpretation accuracy has been found to be difficult in other studies (Mutoro & Wright, 2013) and might be explained by the quality of training or even the experience in practicing interpretation (Ezeofor et al., 2017). Our study was conducted at both hospitals and health centres, and inaccuracy in the interpretation may be due to the lack of familiarity and level of training of the staff. This could be improved by supportive supervision and mentorship so that the staff can improve his or her skills, which has been an effective approach in improving child health services (Manzi et al., 2014). These strategies can also be used to improve the quality of interpretation using paper‐based growth charts, as described in a study in Kenya (Mutoro & Wright, 2013). Lack of improvement in accuracy of the intervention group may have also been related to the design of the mHealth tool. The tool plotted an image of a child on a coloured bar illustrating whether the child was normal (green), moderately malnourished (yellow) or severely malnourished (red). Although the colour‐coded system was helpful for communicating with parents, it could be hard to correctly identify the result with borderline z‐scores because the child icon could span two colour areas. Since this study, we have made improvements in the design of this part of tool by adding numeric z‐scores to the image (see Figure S1). In addition, we have added warning notifications when implausible values have been added to the tool. We hope these improvements will allow users to correctly identify borderline z‐scores and will prevent the errors, which may be related to reading and recording wrong results. Future improvements would include integrating the mHealth tool into the EMR system, which would allow both the measurements entered into the app and the tool and calculations provided by the tool to be stored in the patient's medical record. Creating a more integrated system where the mHealth tool is embedded in the EMR could reduce opportunities for data entry errors, provide more effective digital decision support, improve accuracy and completion of growth classification and seamlessly collect monitoring and clinical data.

This study has some limitations. The two pre‐ and post‐intervention groups are unmatched; however, the population and care providers in both comparison and intervention clinics serve similar populations and had generally the same characteristics. The key difference in the PDC programmes in these two districts during this study period was the extensive decentralization that occurred between the pre‐ and post‐intervention period in the intervention district. Although we cannot rule out that the significantly greater improvements associated with the introduction of an mHealth intervention reflect processes related to this decentralization rather than a true effect of the mHealth intervention, in general, we expect the quality of care delivered at health facilities that are developing new PDC clinics to be not as good as the quality of care delivered at hospitals. Therefore, we do not believe our results can be fully explained by bias due to the concurrent decentralization in the intervention district. We were not able to control other possible interventions from the Rwandan Government such as trainings provided to care providers, which may have differed between the intervention and control districts. However, as we collaborate closely with the facilities, we are not aware of any specific growth monitoring trainings for staff. In addition, we are unable to know whether the mHealth tool was used during PDC visits at the intervention sites as the tool does not track uses or record measurements; however, through qualitative feedback, the tool is very valued by providers, and we assume that it is used most of the time. Additional formal qualitative evaluation feedback may shed more light on this. Lastly, we used data that are routinely documented on patient charts by care providers and then entered into the EMR system, which led to some errors in recording of measurements and missing data. Despite regular data quality assessments by district EMR coordinators and identification and correction of implausible values during the analysis process, we were unable to verify the accuracy of raw measurements of weight, length/height and age that were recorded by the nurses and used to calculate the WHO standard growth classifications in our accuracy analyses.

## 5 CONCLUSION

The introduction of an mHealth tool was associated with significantly greater improvements in the completeness of nutritional assessments and nutrition status of infants at 6 months of age in intervention clinics. Although the accuracy of growth monitoring did not change, these findings suggest that integrating mHealth in providing nutrition interventions is feasible and may help in improving child nutrition outcomes. Further work is needed in improving the intervention with integrated digital systems with mHealth tool connected with EMR system and in improving the design of the tool with precise visibility and determination of z‐score. To reduce the level of inaccuracy, more research is needed to develop good training programmes to improve interpretation of growth charts as well as providing mentorship.

## CONFLICTS OF INTEREST

The authors declare that they have no conflicts of interest.

## CONTRIBUTIONS

MN, ACK, SU and VB designed the study, interpreted results and drafted the manuscript. DAB provided methodologic assistance on study design, conducted statistical analysis and critically reviewed the manuscript. KB and CMK designed the study, supported interpretation of results, analysis and critical reviews of the manuscript. AN, KW, JB, SH, AU and EN contributed to results interpretation and manuscript revision. All authors have read and approved the final manuscript.

## TRIAL REGISTRATION

ClinicalTrials.gov Identifier: NCT03665012.

## Supporting information

**Data S1.** Supporting information

**Figure S1.** Selected screenshots of the PDC mHealth Tool after Improvement

## Data Availability

The data that support the findings of this study are available from the corresponding author upon reasonable request.
