# Supplementary material for: Evaluation of an mHealth tool to improve nutritional assessment among infants under 6 months in paediatric development clinics in rural Rwanda: Quasi‐experimental study
Source: Matern Child Nutr. 2021 May 7;17(4):e13201. doi: 10.1111/mcn.13201 (PMC8476404; doi:10.1111/mcn.13201)
Supplement: Supplementary file 1 — Data S1. Supporting information [file MCN-17-e13201-s001.doc]

**Supplementary Material 1:** A consort diagram

Extracted Total visits from EMR (n= 16,636)

Excluded:

observations with incorrect system IDs (n=56)

Repeated datalines (n=361)

Visits of children not newly enrolled during study period (n=8,732)

Post-intervention visits among children who enrolled in the pre-intervention period (n=3958)

Eligible for analysis (n= 3529)

Comparison clinics (n=1,566)

Intervention clinics (n=1,963)

Pre-intervention period (n=866)

Post-intervention period (n=912)

Pre-intervention period (n=700)

Pre-intervention period (n=1051)
