# Supplementary material for: Evaluation of an mHealth tool to improve nutritional assessment among infants under 6 months in paediatric development clinics in rural Rwanda: Quasi‐experimental study
Source: Matern Child Nutr. 2021 May 7;17(4):e13201. doi: 10.1111/mcn.13201 (PMC8476404; doi:10.1111/mcn.13201)

**Supplementary materials 2:**

**Figure 1. Selected screenshots of the PDC mHealth Tool after Improvement**

***
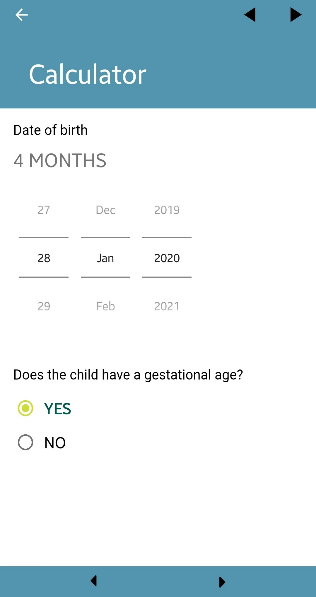
***  *
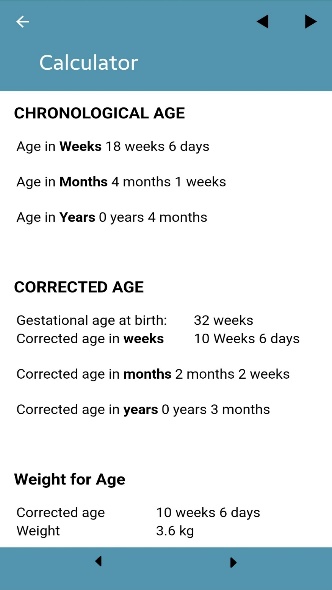
*

##
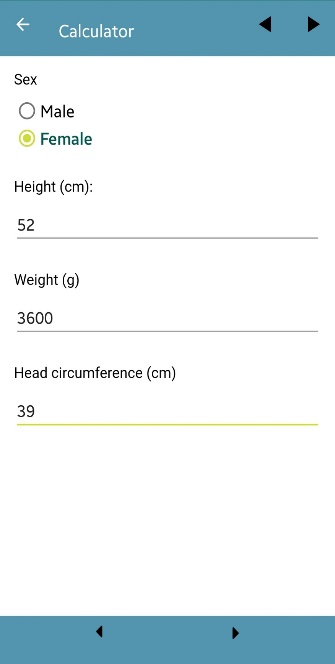

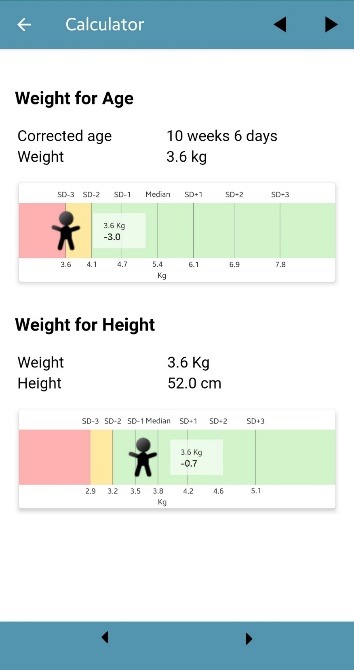


##
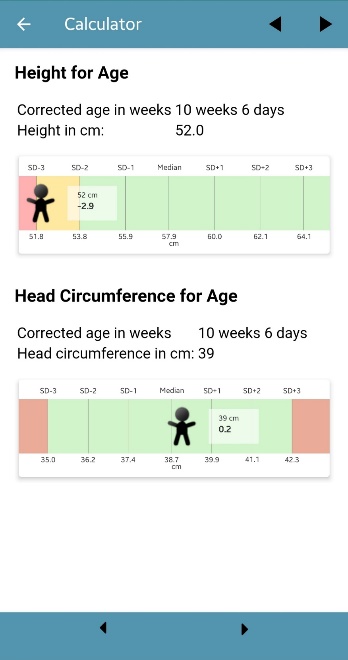

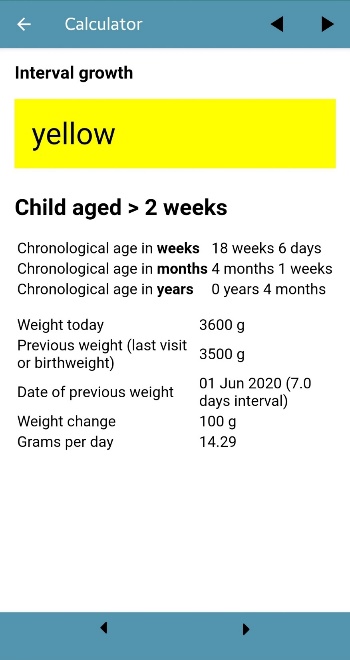

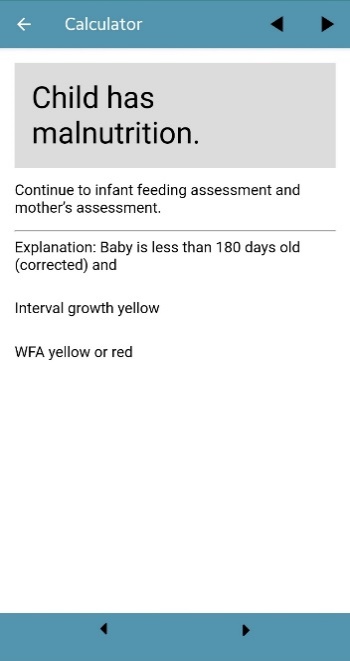

Supplement: Supplementary file 2 — Figure S1. Selected screenshots of the PDC mHealth Tool after Improvement [file MCN-17-e13201-s002.doc]
